# Miscellaneous

**Published:** 1849-07-01

**Authors:** 


### Miscellaneous.

### REMARKS ON THE CAUSES AND MORBID ANATOMY OF MENTAL DISEASES.

BY JOHN WEBSTER, M.D., F.R.S., &c.*

(*From the* LANCET.)

THE author commenced his paper by observing, that having had two of his communications already published in the 26th and 28th volumes of the Society's *Transactions*, on the Statistics and Pathology of Mental Diseases, he presented the present as a further exposition of the subjects then discussed. He then stated, in illustration of the comparative frequency of madness in the two sexes, that out of 1798 lunatics admitted into Bethlem Hospital, during six years, ending the 31st of December last, 1094 were females, and only 704 male patients. He next alluded to the causes apparently producing insanity, which he divided into moral and physical, besides hereditary tendency to mental disease. Of the male lunatics, nearly one half, or 346, became mad from moral causes; whilst the proportion of females was not quite so considerable, being 489 of the entire number. By physical causes, less than one-fourth of the male lunatics, or 156, became insane; whereas, amongst the female patients, the proportion was rather larger, being 282. Hence, speaking generally, moral causes produced half the total cases; but physical causes only one-fourth. The principal moral influence which occasioned insanity amongst males was reverse of fortune, whereby 86 examples are recorded. Next, anxiety, which furnished 69 instances; then religion, giving 45 cases; lastly, love, which caused the loss of reason in 18 men. Amongst female lunatics, anxiety was the most frequent moral cause, producing 79 instances out of the 489 patients classed under the above category; whilst 69 cases arose from religion; 62 from the loss of relatives; and 57 from the more powerful influence of Cupid's tender passion upon the susceptible feelings of women. Fright caused insanity in 50 cases; reverse of fortune in 49; whereas, amongst men, as already stated, the same cause produced nearly treble that amount, speaking comparatively. In regard to physical causes, of the 156 male lunatics so affected, 80 originated from intemperance; and of the 282 female lunatics, similarly classified, 117 arose from puerperal disease. Other physical causes were subsequently mentioned by the author, before passing to the examination of hereditary tendency to mental complaints. Of 704 male lunatics previously enumerated, 219, or 31.10 per cent., had hereditary tendency to mania; but of the 1094 female insane patients, the proportion was larger—namely, 390, or 37.47 per cent. The author, amongst other remarks respecting the influence of hereditary tendency in producing mental disease, said, such an important fact, wherever it exists, should be always well considered by parties forming matrimonial engagements; especially, when both families are unfortunately so tainted. He next adverted to the age at which insanity is most likely to supervene. In males, he stated the most susceptible time of life was from 30 to 40; but in females it was earlier, being from 20 to 30. The author subsequently noticed the two sections of psychological physicians now dividing the profession—namely, the "vitalists" and the "anatomists," of which latter body he is himself a disciple, since he considers their doctrine the most rational, and in a greater degree consistent with the

* Read before the Royal Medical and Chirurgical Society, Tuesday, May 8, 1849, Dr. Addison, president.

present advanced state of pathological knowledge respecting mental diseases. The author afterwards gave a synopsis of sixty-seven dissections made at Bethlem Hospital, of which the following is a summary of the diseased appearances observed in the brain and membranes. In 53 cases, effusion of water had taken place in the ventricles; in 53 cases, also, there was infiltration of the pia mater; in 38, turgidity of the cerebral blood-vessels; in 30, the arachnoid membrane was thickened and opaque; in 26, the colour of the brain was altered from its natural tint; in 15, there was an effusion of blood within the skull, besides other alterations of structure, as mentioned by the author. The organs of the chest were likewise more or less diseased in as many as 62 of the patients; whilst in 30, morbid changes were likewise noticed in the abdominal viscera; so much so, indeed, was this the case, that the immediate cause of death, in a number of the insane patients referred to in the present communication, was apparently disease in these parts; but more especially, affections of the organs of respiration. Dr. Webster then alluded to the long period during which some of the lunatics had laboured under mental aberration, particularly females, one female lunatic having constantly resided in the incurable ward at Bethlem Hospital for upwards of half a century, or actually fifty-four years; thereby showing, that the loss of reason is sometimes not incompatible with longevity. After again referring to the deductions contained in his previous papers communicated to the Society, the author concluded by remarking, that the facts and statements now brought forward, fully confirmed his former observations, and he hoped they might prove useful to students of medical psychology.

Mr. Solly, whilst he complimented Dr. Webster on the value of his paper, could not help regretting that no account had been given of the appearance of the cortical substance of the brain—that portion of the cerebral mass which was essentially connected with the intellectual manifestations. He thought we were in error in our dissections of the brain, to view the organ as one whole; but that we should, as we did with the contents of the chest or abdomen, examine and describe each individual organ and part. Dr. Bright had referred to the colour of the cortical substance of the brain in some dissections of insane patients; and he (Mr. Solly), in examinations at Hanwell and elsewhere, had, as a general rule, found this portion of the brain higher coloured when excitement preceded death, but pale if the insanity had been of long continuance, and without excitement. He (Mr. Solly) had called this portion of the brain the "hemispherical ganglia," for want of a better term, and regarded its appearance after death as most essential to be mentioned in cases of insanity. One interesting fact was mentioned in the paper, and that was, the frequency with which the pia mater and arachnoid were found injected; these only acted in insanity, of course, by influencing the brain in their immediate neighbourhood. He therefore assumed that the brain so situated was affected, as this condition of the membranes was not sufficient to produce the insanity.

Dr. A. J. Sutherland was sure that the Society must feel much indebted to Dr. Webster for bringing under their notice much important information relative to the causes and the pathology of insanity. He did not know any subject so difficult as that which Dr. Webster had chosen. The causes of insanity were in many cases difficult to be ascertained; the friends themselves were frequently mistaken as to the real cause of the illness; and it was only now and then that we were able to correct the mistakes of the friends, from the symptoms of the case. But again there were these difficulties which beset us when we investigate the causes of the disease: what obtains in hospitals, such as Bethlem and St. Luke's, did not always obtain in other places; what prevailed among the patients of one class of society, did not equally prevail among those of another. Thus, the hereditary predisposition to insanity was, as stated in the paper, one-third; as stated by others, one-sixth among the lower classes, whereas it was as much as one-half among the higher. With respect, also, to sex, a greater proportion of females in the lower class, and a greater proportion of males in the higher classes of society, become insane. Therefore we were likely to be misled, if we took any particular class of patients as examples of what prevailed generally; for not only were there differences in the proportion, but there were differences in the species of insanity, in different places. Thus, in the agricultural districts of this country, the proportion of the insane to the sane, was as 1 to 800; while in the manufacturing districts it was as 1 to 1200. But not only the occupation, but differences in climate and diet appeared to have an influence in the production of this or that species of nervous disorder. Dementia and imbecility were supposed to be common in marshy countries; hypochondriasis abounded in Iceland; and in the Western Islands of Scotland, the nostalgia of the Swiss, the cretinism of the Vallais, and the pellagra of Lombardy, were also familiar instances. He differed in some respects with Dr. Webster, as to what he had observed relative to the pathology of insanity, not, he meant, with regard to post-mortem examinations, which formed but a small part of the pathology of any disease, but with regard to pathology in its widest sense, drawn from the symptoms during life, as well as the appearances after death,—drawn from the causes, even from the treatment of the disease. He thought that Dr. Webster had done well in not considering the pathology of insanity apart from its causes; for as there were distinct causes, moral and physical, so there were distinct origins of the disease. The disorder might take its rise primarily from the nervous centres, or the different organs of the body. The liver, the uterus, and the stomach, might affect the brain, and insanity might be the consequence. Insanity, therefore, might be said in one sense to be idiopathic; in another sense, symptomatic; but whether the one or the other, it was the seat of the intellect and the affections which was deranged; and therefore we looked with peculiar interest to the cerebrum, to throw some light upon these difficult investigations. With respect to post-mortem examinations, his experience corresponded with the facts recorded in the paper. He had never found the results of acute inflammation in the brains of lunatics, and what Mr. Solly had stated with regard to the cortical structure was certainly correct—viz., that there is, for the most part, a state of hyperæmia in acute cases, a state of atrophy in chronic cases; there was also a disposition to venous congestion in some brains, to active congestion in others; but the effusion into the sub-arachnoid tissue, and into the ventricles spoken of in the paper, and so frequently found in these cases, was rather the effect than the cause of the disease; in the acute stage it was probably the result of venous congestion, and in the chronic stage it was due to atrophy of the brain, just as the spinal marrow becomes atrophied in tabes dorsalis. But he apprehended there were few who would think that what we saw after death was all that had occurred during the progress of the disease, and therefore the pathologist would be inclined to apply to the symptoms which he had seen during life, and to the analysis of the different fluids of the body, to help him in his investigations. With respect to the former, the symptoms which the intellectual faculties furnished were those of undue activity on the one hand, and of extreme dulness on the other, while the physical symptoms were generally those of irritation, sometimes of congestion, never of acute inflammation. From the analysis of the blood of insane patients, we knew that there was no excess of fibrin, while from the analysis of the urine we obtained sometimes a plus quantity, sometimes a minus quantity of the phosphates, thus confirming, as far as it went, the evidence which we derived from other sources. With respect to the analysis of nervous matter, L'Héritié had shown that there was a minus quantity of phosphorus in the brains of idiots; and Couerbe asserted that he had found a plus quantity of phosphorus in the brains of maniacs. But not only was it a matter of importance for us to ascertain the quality of the blood, but it was also requisite to estimate the quantity which circulated in the brain. Not only had we examples of insanity from the poison of other diseases from bad blood, but we had examples from local congestion of the brain, and from anæmia. Many patients were admitted into St. Luke's, whose disease had originated in low diet and starvation, in whose brains might be supposed to have commenced that process of oxidation which Liebig called "eremacausis." But we should also feel inclined to examine into the condition of that subtle fluid, the nervous force, which ministered to those influences by which the mind manifested its ideas, and which, when disordered, counteracted and obscured its development. These were the heads of some of those subjects which he trusted at no distant period might throw light upon the pathology of insanity.

Dr. Webster said, that in reference to Mr. Solly's remarks respecting the morbid changes in the cortical substance not being sufficiently distinguished from the appearances observed in the medullary; this arose from the fact, that only a summary, not the particulars, of the various autopsies were detailed to the Society. Had the synopsis been read entirely, the points adverted to by that gentleman would have been explained. All the morbid changes noticed were accurately mentioned, and when it was remembered that most of the dissections had been made by so distinguished an anatomist and physiologist as Mr. Lawrence, this must be a sufficient guarantee of their value and accuracy. Dr. Sutherland had alluded to the influence which climate, geographical position, and the civil condition of individuals exerted in the production of insanity. In many of these remarks he fully concurred, and would even affirm that the religious, political, and social status of the inhabitants in particular countries, materially affected the results produced by ordinary exciting causes. For instance, insanity is a much more common disease in cold climates than in temperate or warm countries. In Sweden and Norway a larger proportion of the inhabitants become mad than in any other part of Europe. The disease is more frequent in North Germany than in the southern part of that empire. Mania prevails more in Belgium than France, in the northern departments of which latter country it is met with in a higher ratio than amongst the natives of the south. In Spain the disease is less common than in France; whilst in Northern Italy, insanity is reported to be twice as common as in the southern part of the Italian peninsula. Again, on the southern shores of the Mediterranean, the disease is still less frequent—as in Egypt, Syria, &c.; and in Arabia, mania is so rare, that it is seldom observed; indeed, a chief physician to the great hospital at Alexandria has stated, that during ten years he had only met with one insane Arab, notwithstanding the number of Arabians in Egypt, and the large population of that city. To show the influence which agitation and great mental excitement produce amongst the inhabitants of a country in reference to the disease under discussion, he (Dr. Webster) might mention that madness was frequent amongst the Crusaders, whilst the disastrous effects of this enthusiasm continued long afterwards. During the reformation in Germany, the Low Countries, and in Britain, insanity became common; likewise during the civil wars of England, and the domination of puritanism under the Long Parliament and Cromwell. The first revolution in France caused many cases of insanity, and when Napoleon upset dynasties, made kings, queens, and titled personages, almost by wholesale, imaginary sovereigns and princes were numerous in the asylums of France and Germany, of which Pinel gives examples, and tells us that in Bicêtre there were, at the same time, three Louis-the-Sixteenth maniacs under treatment. As stated in the paper, the recent revolution in Paris had produced most melancholy results on the minds of many individuals of that formerly gay capital. He (Dr. Webster) could say so from his own personal observation, verified by the experience of physicians attached to lunatic establishments, private as well as public, of that country, where political excitement, clubbism, and the late *bouleversement* of public institutions and private fortunes, have occasioned very lamentable consequences. Dr. Sutherland remarked, although more females became insane in the lower ranks of society than men of the same class, still, amongst the higher grades of people in Great Britain, more men than women were affected with madness. This was certainly true to some extent, especially as hereditary tendency to mania appears more prevalent in the upper than the lower classes, and purity of blood, as it is erroneously called, often influences matrimonial engagements to a greater extent than amongst the commonalty. This was formerly well exemplified amongst the old noblesse of France, the clans of Scotland, and the *sangre azul*, or blue blood of the Spanish *grandezza*, in all of whom mental and physical qualities were then transmitted to offspring in greater purity, whether for good or evil, than amongst the more mixed blood of common people. Notwithstanding the facts alluded to by Dr. Sutherland, insanity was generally more common in women than men throughout England, as shown by the recent report of the Lunacy Commissioners, and as the patients treated at Bethlem Hospital were not paupers, but often persons of education, such as governesses, clergymen, merchants, and many others of a similar situation in life, but broken down by disease as well as poverty; and as they come from all parts of the country, are not confined to the metropolis, but often the reverse, the results thus met with respecting the two sexes, therefore, constitute a good criterion, and support the conclusions stated in the present paper. Religious persuasion exerts considerable influence in this malady; at least, it seems to be more common among Protestants than Catholics, and prevails to a greater extent in converts and proselytes, than in persons of confirmed steady faith, whilst it is oftener met with in countries where religious controversies are common, than elsewhere. Many other points might also be mentioned, bearing upon the question of insanity; but although interesting, he would not trespass upon the time of the fellows further than to observe, that the chemical investigations alluded to by Dr. Sutherland were most important, and deserved the attention of practitioners: that physician had already done much to elucidate this subject, and he hoped soon to see more accomplished in the above branch of medical science, as applied to mania. The field was both rich and extensive, and now that the profession generally had begun to study mental diseases, their nature and treatment, in the way they deserved, much benefit would thereby accrue, as well to medical men as, through them, to the community.

Mr. Streeter considered the Society deeply indebted to Dr. Webster for the large amount of information, drawn from the records of Bethlem Hospital, which he had placed before its fellows. No one had taken so much pains as Dr. Webster to make the experience of that institution available to the profession; and his papers certainly afforded valuable materials for the study of insanity. When, however, Dr. Webster called upon medical men to apply themselves to the study of this disease, he begged to inquire where the facilities for following out that study existed? They were certainly not to be found in the general or special hospitals of this metropolis. It might be truly said that means were not afforded to the members of the profession for qualifying themselves for the duties they were called upon to perform in domestic life, and in civil and criminal courts, in the questions that continually arose in reference to insanity. The consequence was, that they were far from being so highly prepared as the bearings of this disease upon many of the most important interests of society rendered desirable. Dr. Webster had certainly omitted much that was required for the complete study of insanity; but when Mr. Solly alluded to his omitting to describe the colour of the hemispherical ganglia, or, more popularly speaking, of the cineritious exterior of the cerebral lobes, knowing the belief which Mr. Solly entertained of this part comprising a series of ganglia, each performing separate and independent functions, and not being a single organ, he felt disposed to ask, why, in the account of his dissections at Hanwell, Mr. Solly had spoken of the cineritious structure generally, and without any attempt to localize its morbid appearances. On several occasions he had seen the morbid appearances of the cineritious structure localized. In puerperal mania of rapid termination, the point of the posterior lobe had acquired a much deeper colour; and in many instances, where the convolutions were carefully unfolded, one after another morbid changes of colour and structure would be detected, that would otherwise escape observation. And here chemical and microscopical investigation promised to be useful. It was, however, in that stage of insanity which was most interesting to the family medical attendant that these communications were most defective. He meant, the approach and incipiency of insanity—that stage in which the disease was most amenable to medicine—that to which Dr. Winslow had given the term of incubation. He had heard, with pleasure, the distinction pointed out by Dr. Sutherland, of centric and excentric cases—a distinction of great practical value, as he believed the excentric, as in other nervous diseases, were more easy of cure. As an illustration of this, he would mention the rapid removal, in many cases, of the excitement and delusions of delirium tremens by the influence of opium on the stomach and intestines. He might also adduce, as proofs, the cases of curable insanity connected with uterine disease, which Dr. Lever had recently brought before the profession. He himself considered that the foundation of intemperance and insanity, in after life, in females, was often laid by the prevailing opinion, that vaginal and uterine discharges always indicated weakness, and a state of system requiring stimulation and fermented liquors. Great caution was required before removing them by local means. He thought Government must, at no distant period, insure the means and enforce the study of insanity upon the profession generally, since all were called upon to perform duties connected with it. In conclusion, he alluded to the set which had for some time past been made against doctors, by the higher grades of lawyers, in reference to generally-received opinions upon insanity. He would say, that if it was the province of their profession to hinder crime from escaping punishment, by simulating insanity, it was the high calling of ours to prevent insanity from being visited and treated as crime.

Dr. Sibson said it was remarkable that in Dr. Webster's paper so few cases of *ramollissement* or of induration had been enumerated. The blood that had been found in some cases, the fluid in others, and in others, again, the thickening of the arachnoid and pia mater, were the most commonly observed changes which were found in cases of diseases in which insanity was not present. He regarded the chemical results as the most definite in this inquiry. But we need not look at the brain only in our investigations into the pathology of insanity. The internal organs required to be examined also. The effects of conformation had their influence in the production of this disease. The condition of the stomach, the uterus, and of the heart, required to be looked to. He considered that at the present moment we were not in a condition to say that we had been able to trace a clear and distinct connexion between the brain and insanity; at all events, all the changes which had been enumerated, might exist in the brains of those in whom insanity had not been present; and when met with in the brains of the insane, he thought they were only the consequences of the insanity. Besides the inquiries into the effects of climate, and the other points mentioned by Dr. Sutherland and Dr. Webster, he thought we should extend our observations to the texture of the skin, the hair, &c. Muscular conformation, the size of the lungs, the power of the heart, the condition of the abdominal organs, and of the digestive apparatus; indeed, the state of every limb and of every fibre in the body constituted an element of inquiry in cases of insanity.

Dr. Webster remarked, as it seemed to be implied from the observations of previous speakers, that he had omitted to notice the symptoms and treatment of mental diseases, he must state, to prevent any misconception of his object in the present communication, that this was done purposely, his remarks being confined to the causes and consequences of the above class of affections. The subject was too extensive to admit of any other proceeding; and in respect of the remarks made by Dr. Sibson as to temperament, physical conformation, colour of the hair, texture of the skin, and so forth, these points had by no means been overlooked by psychologists; on the contrary, great attention had been recently given to similar inquiries, and much valuable information thereon collected. Some physicians have also even shown that the colour of the eyes was important; and M. Foville, recently physician to the asylum at Charenton, but from which he was displaced by the late red republican government, considered the configuration and structure of the external ear as often indicative of insanity. This peculiar appearance of the ear, M. Foville had pointed out to him, (Dr. Webster,) when lately visiting Charenton, and he had since seen the same thing in this country; indeed, it had been noticed by others previously. The observation of Dr. Sibson respecting the importance of studying the connexion of symptoms with the pathological appearances met with after death, could not be over-rated; and although nothing was said in the paper, for the reasons already adduced, there could not be any doubt respecting the advantages of such an inquiry, and he hoped subsequent investigators would supply this deficiency. Reverting again to the causes producing insanity, it might be interesting to state, that intemperance, owing to the improved habits of the people in this country, did not seem to be so frequent an exciting cause of that malady, as formerly; on the other hand, the now prevalent habit of smoking tobacco had very much taken the place of intoxicating drinks; and in America, where intemperance, chewing opium, and smoking, were enumerated amongst the causes producing mental disease, considerable attention had recently been directed to the subject. In his (Dr. Webster's) opinion, this filthy custom was most injurious, as well to body as mind; and whether intoxication was produced by spirits, opium, or by tobacco, all were abominations, and he believed physicians, conversant with mental maladies, were every day becoming more and more convinced of the bad effects arising from this narcotic weed; whilst in some asylums its use amongst the lunatics was strictly prohibited. If he (Dr. Webster) did not farther advert to other points connected with the subject under discussion, it was not through want of inclination, but from the desire to confine his remarks to questions mooted in the present communication. However, as the study of insanity was now assuming the place in medical education which its great importance deserved, he had not a doubt but in due time every information would be supplied by the diffusion of sound practical knowledge.

---

### THE NEW COUNTY ASYLUM FOR MIDDLESEX, COLNEY HATCH.

The crowded state of the Hanwell Asylum, the daily applications for the admission of new patients, which are necessarily refused, the number of pauper lunatics scattered through the different workhouses of the metropolis and its neighbourhood, have at length rendered the erection of another asylum for the county of Middlesex no longer a matter of choice, but of imperative necessity; for although several parishes somewhat selfishly held meetings and drew up petitions against the proposed measure, which they objected to on account of the increased assessment of the county rates thereby rendered necessary, still the cause of humanity has triumphed, and the foundation-stone of the Colney Hatch Lunatic Asylum was laid on the 8th of May, by his Royal Highness Prince Albert, in the presence of the Lord Mayor of London, the Lord-Lieutenant of the County, a numerous body of magistrates, and an august assemblage of noblemen and gentlemen.

The site selected for the new asylum is within a mile of the village of Southgate, at Colney Hatch, within the parish of Friern Barnet, about ten miles north of the metropolis. The situation is airy and healthful, the surrounding scenery cheerful, and perhaps, as beautiful as may be found in any part of the county; it will also have the advantage of being adjacent to one of the stations of the Great Northern Railway, which is now in course of construction. The building, we are informed, will be in the modern style of Italian architecture; and it is expected that it will be completed, and ready for the reception of lunatics, by Michaelmas, 1850.

The attention which has during the last few years been paid to the subject of lunacy—the psychological and pathological investigations which have only recently rendered this study a recognised and legitimate branch of medical science—the improvements which have been introduced in all the practical details of the treatment of the disease—and especially the opportunities we now enjoy of comparing many admirable institutions of the kind with each other both at home and abroad—these, and a variety of other considerations, induce us to expect that this new asylum will be built upon the most approved principles, and that it will combine within its walls all the advantages which our present advanced state of knowledge and practical experience can command. Not only do we apply these remarks to the architectural structure and plan of the building, but to its internal organization, medical and moral. And here we are struck, *in limine*, with one important fact—the average number of pauper lunatics in the county of Middlesex is stated to be 2400, and the disease is with the population notoriously progressive. If, therefore, 1000 patients be located at Hanwell, and accommodation be provided at Colney Hatch, even for the remaining 1400, there will still, it is to be feared, be in a very few years, a surplus number of pauper lunatics encumbering their respective parishes. We entertain, therefore, some doubt whether such enormous establishments are expedient, and whether district lunatic asylums, capable of holding 500 patients each, would not be more desirable. Hanwell was, in the first instance, designed to admit only 300 patients; the building was afterwards extended, at an enormous expense, so as to enable it to receive the present number of inmates; and we feel very much inclined to predict that a similar fate, notwithstanding the extent of the accommodation contemplated, will await Colney Hatch Asylum. It occurs also to us that there are many objections to so vast a number of patients being congregated under one roof. The extreme length of the new building, we are informed, is to be 1883 feet 6 inches; its extreme breadth, in the centre, 143 feet 10 inches; and there will be wings on the right, for females, in length 586 feet 6 inches; and on the left, wings for men, in length 500 feet 6 inches. We are well aware that the male will be separated, as at Hanwell, from the female department; but we cannot help thinking that it would have been better for each sex to have been located in a distinct and separate building. If the Commissioners in Lunacy find it expedient, for moral considerations, not to license any more houses for male and female patients of the better classes conjointly, but insist that such establishments shall be appropriated exclusively to the one or other sex, how much more reason, *à fortiori*, is there for having pauper male and pauper female lunatics located in separate asylums? We know that notwithstanding the interposition of walls between the two departments in these large buildings, irregularities are said to arise from having the two sexes under one roof.

One of the great inconveniences in Hanwell is the great circular stone staircase in the centre of the building, and other staircases and steps, which are, for many reasons, clumsy, if not dangerous, which will, it is stated, be avoided in the new asylum, by the basement story being approached by a corridor going down to it upon an inclined and level plane, which will supersede the necessity of steps. The proportion also between single cells and dormitories should be well considered. There are certain cases in which the separate cell system is advantageous to the patient; but it should not be forgotten that solitude, even during the hours of night, is in many other cases very objectionable. The disposition of the lunatic upon the invasion of his malady, is generally to be anti-gregarious; and to make him associate with his fellow-men, and draw his mind out of the cloudy solitariness in which it preys upon its own delusions, is one of the most obvious indications of cure. Besides which, during the silent hours of night, in the solitary cell, patients will often indulge in vices which the presence of their fellow-patients in a dormitory may check. We do not, of course, refer to inveterate cases, in which every mode of control is sometimes set at defiance; but we affirm that the single cell system, in the beginning of this malady, is favourable to the indulgence of this morbid propensity, at a time when the moral sense of doing wrong is susceptible of being influenced by the presence of those around, and that a dormitory is therefore preferable for such patients. It is alleged, we are aware, that the atmosphere must in every dormitory become, during the night, impure, but all we can say is, they "manage these things better in France;" if the ceiling be lofty, and the ventilation good, no such unpleasant consequences would arise. Moreover, it is well observed by the Commissioners in Lunacy in their Report in 1844, that the dormitories in many of the county asylums "accord better with the pauper's previous habits than sleeping alone in a solitary cell, with a single window; and the companionship of others in the same room does not seem to interfere with their nightly rest. We rarely (they continue to observe) visit a licensed house without asking some of the patients how they sleep at night, and we are generally answered that they sleep well. These persons almost invariably occupy sleeping-rooms containing several beds. In many good licensed houses, also, private patients of a superior class frequently sleep, to the number of four or five, or even more, in separate beds, in the same room. Upon the whole, we are of opinion that dormitories containing several beds are much preferable as a general arrangement to cells or single-bedded rooms, although a *limited* number of the latter is doubtless necessary in every large asylum for the use, amongst others, of violent, noisy, and mischievous patients, and for such as are labouring under a paroxysm." Our own experience and observations accord with these remarks of the Commissioners; besides which, it might be added, that dormitories are the safest for suicidal patients, with whom the constant presence of companions has a sanative effect.

While it is promised that the external aspect of the new asylum will present us with a cheerful Italian style of architecture, the internal walls will not, we hope, be permitted to remain so dead and prison-like as those we find at present in our public asylums. The plain whitewashed surface surely would admit of some little decoration or relief, even by being papered, as also the corridors and passages, which are as blank and cheerless in many asylums as those in the interior of Newgate itself. There is another and a still more important object which, we trust, will be kept steadily in view. The dreary interior of these buildings seems to accord well with the notion that their unfortunate inmates are inaccessible to happier impressions and associations from surrounding objects, and that they therefore are consigned as absolutely incurable to the

> "Vast lazar-house of many woes,
> Where laughter is not mirth, nor mirth the mind,
> Nor words a language, nor even men mankind."

But we would fain hope that these gloomy days have passed away, and that the new county asylum of Colney Hatch will be essentially a *curative* institution, and provided above all things with an efficient medical staff. Indeed, now that the progress of mental and cerebral pathology has clearly demonstrated that insanity is, in its early stages, as curable as any other disease incident to humanity, we anticipate that, with the advantages which this asylum can command, it will soon acquire an European reputation. With this anticipation in our mind, we return to the imposing ceremony, the details of which it is our pleasing duty to record as a matter of history.

On the 8th May, 1849, his Royal Highness Prince Albert arrived at Colney Hatch, a few minutes after twelve o'clock, and was received by the Lord Lieutenant of the County, the Marquis of Salisbury, the Lord Mayor, Sir James Duke, the county magistrates, Benjamin Rotch, Esq., and Henry Pownall, Esq., and a large number of the county magistracy, and clergymen and gentlemen of the district. A procession was then formed, and the Prince was conducted to his seat opposite the stone. On raised benches around him were a large number of ladies and gentlemen, who gave his Royal Highness a most enthusiastic reception. It may be stated, that the foundation-stone of the new institution has one peculiarity, which is, that instead of being hidden from sight, it will henceforth form one of the principal features in the entrance hall. It is a large piece of freestone, having on its front a magnificent sculptured marble entablature, with the following inscription:—

"This foundation-stone was laid by Field-Marshal his Royal Highness Prince Albert, K.G., her Majesty's Consort, on the 8th day of May, A.D. 1849, and in the 12th year of the reign of her Most Gracious Majesty Queen Victoria. May God bless this work of charity.

"Committee of Visitors.—Benjamin Rotch, Esq., chairman; Lord Robert Grosvenor, M.P.; James Bentley, Esq.; J. H. Bluck, Esq.; C. S. Butler, Esq.; E. H. Chapman, Esq.; C. H. Cottrell, Esq.; J. W. Freshfield, Esq.; John Garford, Esq.; T. B. Herring, Esq.; M. H. Kempshead, Esq.; Henry Pownall, Esq.; Hector Rose, Esq.; John Simpson, Esq.; Arthur Smith, Esq.; Edward Stock, Esq.; C. B. Stutfield, Esq.; J. B. Walesby, Esq.; Henry Warner, Esq.; John Wilks, Esq.; and Joseph Wilson, Esq.

"J. S. Scaife, clerk to committee; S. W. Daukes, Esq., architect; Mr. George Myers, builder; C. J. Shoppee, clerk of the works."

The usual preliminaries having been settled—

Mr. B. Rotch addressed his Royal Highness in the following terms:—As chairman of the justices entrusted by the court of quarter sessions with the erection of this asylum, your Royal Highness will perhaps allow me to say a few words, and to draw your Royal Highness's attention to the vast extent of foundations, comprising the officers' residences, the airing courts, the workshops, and the chapel, now lying maplike before us. When completed, it will be the largest lunatic asylum in the United Kingdom, and will be capable of accommodating upwards of a thousand patients. It must not be supposed that the necessity for this additional asylum has arisen from any increase in the malady itself, disproportioned to the increasing population of Middlesex; far from it. It has only become necessary in order to relieve the rate-payers of the county from the heavy burden imposed upon them by the extra expense they are now put to by being compelled to keep more than 1000 pauper lunatics in private asylums, at a cost so greatly exceeding what they would be kept for in a county asylum, that when the thirty years over which the cost of this building will be divided, have expired, the saving will have exceeded the expenditure by several thousand pounds, and every year afterwards will secure a very large annual saving to the rate-payers of the county. It will be gratifying to your Royal Highness to hear that what were formerly the horrors of lunatic asylums will be totally unknown here. None but moral restraints will here be imposed; the ray of reason's light, however small, which it ever pleases the Almighty to leave to these poor sufferers, even in the worst phases of their malady, will here be tenderly cherished, and made sufficient to guide them in peace and gentleness in their path, however darkly shadowed by the clouds of their erring intellect, while that system, first carried out in its fullest extent at Hanwell, (the non-restraint system,) at once the pride and boast of our metropolitan county, will have facilities afforded to it here, from the experience of the committee, the talent of the architect, and the extent of the grounds, which, we trust, will enable us still further to develop its efficiency and its humanity; to draw the veil of oblivion over the melancholy times that are passed, to avail ourselves of all the real improvements of the present day, and to look forward, under Divine blessing, to secure happier results in times to come. Such will be the institution raised in a few short months (owing to the activity and energy of our contractor) of which your Royal Highness is now about to lay a foundation-stone; but before doing so, I am charged to convey to your Royal Highness (and I can only regret my inability to do it in adequate terms) the humble and grateful thanks of the committee of visitors, whom I have the honour to represent this day as their chairman, and also of my brother justices of the county of Middlesex generally, for your Royal Highness's gracious condescension in thus giving the high sanction of your countenance to our labours, by aiding us with your own hands to raise up this mighty building—a monument for future ages of the philanthrophy of the age we live in, and of your Royal Highness's great condescension—a building which will, when completed, be entirely devoted to the great cause of Christian charity and humanity.

His Royal Highness Prince Albert replied (addressing Mr. Rotch) in the following terms: I thank you, sir. I trust you will find that the excellent system you are about to adopt may prove successful. The greatest credit is due to you and the other magistrates for your noble exertions.

Mr. H. Pownall, (the Chairman of the Sessions,) in presenting to his Royal Highness the current coins of the present reign, which were closed in a glass receptacle hermetically sealed, said: May it please your Royal Highness, we hail your Royal Highness on this most auspicious occasion with every feeling of respect and affection, not only in consideration of the exalted rank enjoyed by your Royal Highness as consort of our beloved Sovereign, but also as desiring to evince our sincere admiration of the great liberality and untiring zeal displayed by your Royal Highness in promoting and encouraging all those institutions and undertakings which have for their object, the moral, social, and religious elevation of this great empire. To the county of Middlesex belongs the honour of having already provided an asylum for 1000 of the afflicted lunatic poor, and we are met to-day, under the encouraging circumstance of your Royal Highness's presence, further to carry into effect a wise and enlightened enactment of the imperial legislature, which has enacted that every county in England and Wales shall provide an asylum for their lunatic poor. This law, so wisely framed and so benevolently intended, has not been carried into effect with that zeal and alacrity which the urgency of the occasion seemed to demand. The magistrates of the metropolitan county rejoice this day to see your Royal Highness aiding them in this work of mercy, and cheering them onward in the path of benevolence. They rejoice not only in the encouragement afforded by your presence here this day to themselves, but for the zeal which it is likely to diffuse through the magistracy of other counties who have not yet provided an asylum for their afflicted poor. We have reason to rejoice in the zeal manifested by your Royal Highness for the advancement of science and learning in our country, for the interest you take in all that concerns our commercial greatness, and for the efforts made in behalf of those institutions whose object is to mitigate the sufferings of the poor. We have rejoiced in similar benevolent and enlightened efforts made by princes of the royal house on former occasions, but it remained for your Royal Highness to stretch the cords of Christian sympathy from the palace to the abode of the afflicted labourer, and prove, by a generous and personal service, that there is no class of her Majesty's subjects who do not share your regard and derive benefit from your enlightened and well-directed efforts. The magistrates of this county have for years conducted the largest lunatic asylum for paupers in the country, and, with the assistance of able and willing officers, have introduced a system of management which has called forth the approbation of some of the most eminent medical men in Europe, and which has divested this afflicting malady of the mind of all those cruel and brutal usages which formerly characterized the treatment of the insane. No longer coerced with leg-locks and chains, the patient becomes calm, and if not restored to perfect tranquillity, is left free from mechanical restraint. May the conductors of this asylum far surpass the happiest results which have followed the labours of their brethren at Hanwell, and so long as this building shall last, may this act of your Royal Highness be remembered with gratitude, and when time shall have closed upon us and your Royal Highness, may your robes be as white, and your palm as verdant, and your crown as radiant as those who are now enjoying their eternal felicity above. (Loud cheers.)

Prince Albert (taking Mr. Pownall by the hand): Mr. Pownall, I am much obliged to you for your excellent address. I hope the institution may prosper, and that the admirable system of which you have spoken may work in your new institution to your perfect satisfaction. (Loud cheers.)

His Royal Highness then deposited the coins which Mr. Pownall had handed to him; and immediately afterwards some specimens of standard weights and measures were handed to him by Mr. Cottrell.

The Rev. G. H. Thompson, M.A., Rector of Friern Barnet, having invoked a blessing on the work,

His Royal Highness laid the stone, with the usual formalities.

The Marquis of Salisbury, Lord-Lieutenant of the County, said he had received her Majesty's commands to announce that her Majesty felt the greatest interest in the success of the institution, and the fullest confidence that the results would be of a most important and beneficial character. (Cheers.) Her Majesty had also commanded him to say, that she would commence a fund for the relief of those who were discharged from that institution cured. (Renewed applause). He was sure the magistrates present knew how much benefit had been derived from such institutions in different parts of the country; and one of the principal features in such asylums was a fund such as that her Majesty had expressed her intention of setting on foot. Her Majesty the Queen Dowager commenced a similar fund at Hanwell, and her Majesty Queen Victoria munificently contributed to it. They would now have a fund in their new institution, and he had no doubt it would be a useful one, to be called, "The Victoria Fund."

It is calculated that by the erection of this asylum, the county will save at least 7000*l.* per annum in its provisions for pauper lunatics.

The police arrangements, under the direction of Mr. Inspector Beckerson, were perfect. During the whole of the proceedings, although so many thousands of persons were present, there was not the slightest sign of confusion.

Thus terminated the proceedings of a day which we hope will be not less memorable in the history of the county in which it took place than in the history of the science, which such an institution is, we trust, destined to advance.

---

### THE STUDY OF MENTAL DISEASES.

In previous numbers of our Journal, allusions were made to recent improvements at Bethlem Hospital, towards facilitating the acquirement of practical knowledge respecting the nature and treatment of insanity. When adverting to this important department of medical science, we stated that the authorities of the above-named noble charity had resolved to render the ample means at their command of more use to the profession than hitherto, by admitting medical students to attend the physicians' practice, when visiting the insane patients, on the payment of a moderate fee, which was also to include clinical lectures. Respecting the working of the new measures now in operation, it may be interesting to transcribe from the Physicians' Report for 1848, recently printed, and circulated among the governors, the following paragraph relating to the medical pupils admitted last year at Bethlem Hospital:—

"There has been a considerable increase of attendance and much intelligence displayed on the part of the pupils. The proposed formation of a more regular school at Bethlem will accomplish all that can reasonably be expected; and it is distinctly understood that the physicians will deliver a course of lectures during the spring season of each year, commencing as shall be most agreeable to the wish of the governors; and they will have great pleasure in exerting their best endeavours to render the lectures effective."

During the current season, we understand the attendance of pupils has been more numerous than previously; that Sir A. Morison has delivered several interesting clinical lectures; and further, that Mr. Lawrence will also aid this movement for the promotion of so useful a branch of medical education, by giving three lectures on the pathology of mental diseases, illustrated by dissections made at the hospital, which cannot but prove most valuable, seeing that eminent surgeon is both an accomplished physiologist and an excellent anatomist. Next year, we trust Dr. Monro, the senior physician, who has had great experience on the subject of mania, will also give clinical lectures, in the same manner as his colleagues have done recently; in which case, the profession cannot then say the medical officers of Bethlem Hospital have not fulfilled their promise, or that students do not possess ample opportunities of now obtaining information and experience respecting diseases of the mind; more especially, as not only that institution, but likewise St. Luke's Hospital, are open to pupils, where Dr. Sutherland is most zealous in imparting information to the gentlemen in attendance.

Besides the above means now available for studying insanity, Dr. Conolly, both at Hanwell and at the College of Physicians, has, by his admirable lectures, materially contributed towards the same desirable object; and if the examining medical corporations of Great Britain would require all applicants for their diplomas to possess a competent knowledge of diseases of the mind, as they do at present in regard to those affecting the body, great progress would be then made, by all ranks of the profession, in the study of medical psychology. If this were the case, no practitioner need ever be unwilling to undertake the treatment of insane patients, still less should he feel the least difficulty when called before a jury, empanelled by a writ "de lunatico inquirendo," there to be severely cross-examined by an astute member of the long robe, or even badgered by a counsel experienced in such matters. This occasionally happens; and it is well known that legal gentlemen are often delighted if they can puzzle a medical witness; nay, they consider it quite fair, if made to give contradictory evidence regarding the afflicted subject of inquiry. When medical practitioners have, however, practically studied mental diseases, such exhibitions will seldom or ever occur; at the same time, whilst psychological science is thus promoted, philanthropy will be extended, and a very unfortunate class of the community must thereby be materially benefited.

